# Association of Internet gaming disorder symptoms with anxiety and depressive symptoms and substance use: an international cross-sectional study

**DOI:** 10.1186/s43045-022-00180-6

**Published:** 2022-02-14

**Authors:** Julius Burkauskas, Inga Griskova-Bulanova, Ana Đorić, Yatan Pal Singh Balhara, Arya Sidharth, Ramdas Ransing, Tuong –. Vi Vu Thi, Truong Ngoc Huong, Helin Yilmaz Kafali, Gamze Erzin, Zahir Vally, Mita Rani Roy Chowdhury, Pawan Sharma, Rabi Shakya, Paulo Moreira, Sara Faria, Isa Multazam Noor, Luís Antônio Monteiro Campos, Anna Rebeka Szczegielniak, Dejan Stevanovic

**Affiliations:** 1 Laboratory of Behavioral Medicine, Neuroscience Institute, Lithuanian University of Health Sciences, Vyduno al 4, Palanga, Lithuania; 2 Department of Neurobiology and Biophysics, Vilnius University, Vilnius, Lithuania; 3 University of Rijeka, Rijeka, Croatia; 4 Behavioral Addictions Clinic (BAC), Department of Psychiatry and National Drug Dependence Treatment Center (NDDTC), All India Institute of Medical Sciences (AIIMS), New Delhi, India; 5 Centre for Addiction Medicine, National Institute of Mental Health and Neuroscience, Bengaluru, India; 6 Department of Psychiatry, B K L Walawalkar Rural Medical College, Sawarde, Maharashtra India; 7 South Vietnam HIV Addiction Technical Transfer Centre - University of Medicine and Pharmacy, Ho Chi Minh City, Vietnam; 8 Faculty of Public Health, University of Medicine and Pharmacy, Ho Chi Minh City, Vietnam; 9 Ankara Children’s Hematology and Oncology Training and Research Hospital Department of Child and Adolescent Psychiatry, Ankara, Turkey; 10 Diskapi Training and Research Hospital, Ankara, Turkey; 11 Department of Psychology & Counseling, United Arab Emirates University, Al Ain, United Arab Emirates; 12 United Nations Department of Safety and Security, Cox’s bazar, Bangladesh; 13 Department of Psychiatry, Patan Academy of Health Sciences, School of Medicine, Lalitpur, Nepal; 14 Universidade Lusíada Norte (Porto); Centro de investigação em Psicologia para o Desenvolvimento (CIPD), Porto, Portugal; 15 Psychiatry Department, Dr Soeharto Heerdjan Mental Hospital Jakarta, Jakarta, Indonesia; 16 Universidade Católica de Petrópolis Rio de Janeiro, Rio de Janeiro, Brazil; 17 Department of Psychiatric Rehabilitation, Department of Psychiatry and Psychotherapy, Medical University of Silesia, Katowice, Poland; 18 Clinic for Neurology and Psychiatry for Children and Youth, Dr. Subotic 6a, Belgrade, 11000 Serbia

**Keywords:** Problematic gaming, Gaming addiction, Gaming disorder, Psychiatric symptoms, Substance abuse, Anxiety, Depression

## Abstract

**Background:**

Problematic Internet gaming is an increasingly recognized global mental health problem. This multicultural cross-sectional study examined the association between Internet gaming disorder (IGD) symptoms and anxiety and depressive symptoms and substance use within a sample of young Internet users. In total, 3529 college/university students (1260 (35.7%) males; mean age 21 ± 3 years) were surveyed online. We assessed online gaming patterns using the Internet Gaming Disorder Self-report for College/University Students (ICMH-IGD), symptoms of depression using the Patient Health Questionnaire-9, and symptoms of anxiety using the Generalized Anxiety Disorder scale-7.

**Results:**

IGD symptoms were associated with symptoms of depression, anxiety, and substance use, independent of time spent online, psychiatric diagnosis, culture, or sociodemographic characteristics. For males, more significant IGD symptoms were associated with more extended Internet browsing per day time and higher levels of anxiety and depressive symptoms, while for females, with more extended Internet browsing per day time, marihuana use, and higher levels of depressive symptoms.

**Conclusions:**

Our study found that more overt symptoms of IGD were associated with higher levels of anxiety and depressive symptoms and substance use. Still, these associations differed among males and females, suggesting that gender differences should be considered when planning specific treatments.

**Supplementary Information:**

The online version contains supplementary material available at 10.1186/s43045-022-00180-6.

## Background

Problematic Internet gaming is an increasingly recognized global mental health problem [1–3]. In 2013, Internet gaming disorder (IGD) was included in the Diagnostic and Statistical Manual (DSM-5) as a candidate disorder requiring future research [4], while the International Classification of Diseases (ICD) 11th edition recognizes gaming disorder as a new category which includes online gaming as well as offline gaming [5]. IGD is mainly defined as a condition characterized by an increased priority given to gaming, which takes precedence over other life interests and daily activities, and results in impaired control and continuation of/or increase in gaming despite its negative consequences [6]. These core features of IGD resemble those observed in other behavioral addictions, such as substance-related disorders [7, 8]. Although there is an increased interest in IGD research and even treatment approaches are suggested [9–11], large-scale cross-national studies evaluating possible contributors to IGD symptoms are scarce, and many of them focus on adolescents [12] or children [13]. However, it has been shown that young adults are especially vulnerable to IGD [14, 15]. Therefore, studies aiming at the identification of factors affecting the problematic online gaming in young adults are needed to promote better recognition of individuals at risk for IGD [12, 16, 17] and, as a consequence, faster interventions [18] and better management of symptoms related to IGD, especially facing possible mental health disturbances due to COVID-19 pandemic [19].

Depressive and anxiety symptoms have been linked to poor outcomes across different individuals engaged in problematic Internet use [20]. Recent studies have also suggested that IGD may be closely associated with depression, based on the overlapping neural mechanisms in depressed individuals and those with IGD [21–23]. For example, a community health survey of 190,066 participants conducted in Korea in 2017 found that IGD is associated with depression, impairments of daily activities, reduced quality of life, and suicidal ideation and suicide attempts [24]. Similarly, another Korean study comprising 7200 survey participants found that depression was a common comorbid condition among individuals with IGD and was associated with severe clinical phenomenology, including worsened symptoms of alcohol and nicotine addiction and a generalized anxiety disorder [25]. Indeed, other psychological conditions such as generalized anxiety disorder have also been a common comorbid disorder in IGD [26]. Another study comprising a representative sample of 1531 teenagers and young adults found associations between IGD and higher depressive and anxiety symptoms [27]. On a different note, IGD is often associated with various substance use disorders. For example, a recent review suggested that adults with disordered gaming frequently show problematic or disordered substance use [28]. In addition, several studies analyzing neuroanatomical changes in the brain, including reward and executive functioning mechanisms, have reported similar changes in both IGD and substance use [24, 29–32].

Finally, a notable aspect of IGD studies is gender differences. A recent meta-analysis on gaming patterns in 82,440 participants across 21 countries has confirmed that higher levels of IGD exist in males than females [33]. This might result from several reasons, including differences in neural mechanisms related to craving [34], temperamental [35], or socio-cultural factors [36], and coping strategies [37]. While many studies report gender differences in IGD symptomatology, their relation to possible comorbidities is still unknown and requires further investigation. For example, a survey of 429 French online gamers (mean age 21 years) found that in male subjects, high anxiety and depression scores were associated with IGD, while for females, only depression scores were associated with IGD [38].

Further studies are needed to understand better the interplay between symptoms of anxiety, depression, and substance use in IGD, taking into account gender differences. Thus, this study aimed to investigate the relationship between depression, anxiety, and substance use with IGD symptoms, using an international, multicultural sample of Internet users who completed an online survey. Considering previous studies, we hypothesized to find gender differences in IGD symptoms. In addition, it was expected that depression and anxiety symptom severity would be associated with more significant IGD symptoms. Finally, we had an exploratory aim to investigate the association of substance use with IGD symptom severity.

## Methods

### Participants

We used the data for the present study from an already available database [39, 40]. The data were collected from students pursuing various graduation courses in colleges and universities across 15 countries, throughout the International Child Mental Health Study Group (ICMH-SG; http://www.icmhsg.org), including Bangladesh, Brazil, Croatia, India, Indonesia, Italy, Poland, Portugal, Nepal, Nigeria, Serbia, Sweden, Turkey, Vietnam, and the United Arab Emirates (UAE). The lead authors from different countries were responsible for advertising the study in the respective countries, soliciting the students directly or via students’ organizations, and sending a link to the survey. It was considered a mode of convenience sampling and the engagement rate was not monitored. Before completing the survey, each participant was formally asked to provide online consent. No incentives were given upon completion.

An online survey using a cross-sectional design was considered for the data collection due to its benefits of accessing larger sample pools and reaching heterogeneous groups, its cost-efficiency, and its practical advantages for researching behavioral addictions [39–41]. The online survey with information about the study (i.e., objectives and assurances of anonymity and confidentiality) and the instruments (see below) was made available from February 2018 until June 2019 using Google Forms. The survey questionnaire was prepared and made available in additional languages apart from English for the data collection in the countries (i.e., Croatian, Indonesian, Polish, Portuguese, Serbian, Turkish, and Vietnamese, respectively) for students unfamiliar with the English language. For additional details on the sampling and procedures, the reader is referred to [39, 40].

The present study received approvals from the ethics committees of all relevant countries and conformed to the principles outlined in the Declaration of Helsinki.

### Instruments

The online survey included the following instruments. Sociodemographic information, as well as information on substance use and previous psychiatric diagnoses, was collected first. Next, substance use was assessed with a single question on particular substance use (e.g., alcohol, marihuana, and others) over the past month on a 5-point Likert scale from 0 “no usage” to 5 “using substance almost every day.”

#### The International Child Mental Health Study Group Internet Gaming Disorder Self-report for College/University Students (ICMH-IGD)

The ICMH-IGD is a self-report instrument developed and validated by the International Child Mental Health Study Group to assess problematic online gaming activity [40]. The instrument comprises 11 items rated on a five-point Likert scale (i.e., one “never” to five “always”), where a higher score indicates a greater difficulty. The scale development adheres to the main requirements for measuring IGD [42]. For example, the scale covers all DSM-5 IGD criteria and has good validation sample quality, indicated by psychometric properties evaluated in nationally representative samples. The sum score of the instrument ranges from nine to 45, with higher scores indicating a greater intensity of IGD symptoms present. The instrument was previously validated for the English, Croatian, Polish, Portuguese, Serbian, Turkish, and Vietnamese languages, with good psychometric characteristics demonstrated [40]. The Indonesian version was also psychometrically evaluated and its Cronbach’s *α* was 0.88. The Cronbach’s α of the instrument in the current sample was 0.92.

#### Generalized Anxiety Disorder scale-7 (GAD-7)

The GAD-7 a self-report instrument, comprises seven items scored from zero to three, with total scores ranging from zero to 21 with a higher score indicating greater anxiety symptoms severity [43]. In addition, the GAD-7 instrument has solid internal and test-retest reliability and construct validity [43]. The Cronbach’s *α* of the GAD-7 in the current sample was 0.90.

#### Patient Health Questionnaire-9 (PHQ-9)

The PHQ-9 is a self-report instrument developed for fast and efficient psychiatric screening for depression symptoms [44]. The instrument includes nine items on depression severity scored from zero to three, with total scores varying from zero to 27, where higher scores indicate more significant depressive symptomatology. The Cronbach’s α of the PHQ-9 in the current sample was 0.87.

### Statistical analysis

We expressed qualitative data as percentages and quantitative data as means ± standard deviation. The following substance use groups were considered: depressant drugs (i.e., sedatives or tranquilizers, heroin, painkillers, marijuana, and inhalants or solvents), stimulant drugs (i.e., cocaine, methamphetamines, and other stimulants), hallucinogen drugs (i.e., hallucinogen and club drugs), and alcohol. For all variables, we performed normality tests, including skewness, kurtosis, and one-sample Kolmogorov–Smirnov tests, and found no violations of the normal distribution. Differences in sociodemographic, depression, anxiety, substance use and gaming characteristics of participants, stratified by gender, were tested using independent sample *t*-tests, the Pearson chi-square test, and in cases where the *p*-value was lower than .05 difference the between proportions test. Next, we performed multiple linear regression analyses to determine whether depression, anxiety symptom severity, and substance use were independently associated with IGD severity after adjustment for sociodemographic factors, previous psychiatric diagnosis, culture (countries were coded as dummy variables), and time spent online (i.e., number of hours per day). Thus, we designed five separate multiple regression models, including symptoms of depression, anxiety, and substance use variables (i.e., the PHQ-9, GAD-7, type of substance) as predictors of IGD symptoms severity. We examined scatterplots of residuals to check the assumptions of the regression analysis: normality, linearity, and homoscedasticity. The variance inflation factor and tolerance statistic indicated no problem with multicollinearity; all variance inflation factor values were < 2.4. We performed Bonferroni corrections for multiple regression analyses to reduce the likelihood of type I error. We pre-assigned a *p*-value as the threshold for significance by adjusting the *p*-value for the number of statistical comparisons:

$$p-\mathrm{value}\ \mathrm{of}\ 0.05\div \mathrm{number}\ \mathrm{of}\ \mathrm{multiple}\ \mathrm{regression}\ \mathrm{models}$$

Hence, the corrected *p*-value was 0.05 ÷ 5 = 0.01

We required *p* < 0.01 for a predictor to be considered as significant.

Structural equation modeling (SEM) examined the relationships between alcohol abuse, hallucinogenic drugs, stimulant drugs, depressant drugs, depressive, anxiety, and IGD symptoms. Three models were performed to evaluate those relationships in all the collected and male and female samples. We used the SPSS 17.0 for Windows statistical package (SPSS Inc., Chicago, Illinois) for all analyses and software AMOS.

## Results

Data from 3529 subjects (1260 (35.7%) males; mean age 21 ± 3 years) were available. As demonstrated in Table 1, significant differences were found between males and females in the respective instruments for IGD, anxiety, depression, and the frequency of marihuana and other reported substance use. In total, 79 (2.3%) individuals reported using stimulants, 202 (5.8%) sedatives or tranquilizers, 977 (27.8%) alcohol, 195 (5.5%) marijuana, 67 (1.9%) cocaine/crack, 63 (1.8%) club drugs, 59 (1.7%) hallucinogens, 55 (1.6%) heroin, 66 (1.8%) inhalants or solvents, 64 (1.8%) methamphetamines, and 595 (16.8%) painkillers (details available in a supplementary file).

**Table 1:** Characteristics of all participants

Characteristics	Participants			*P*	Difference between proportions, %
	All (*N* = 3529)	Males (*n* = 1260)	Females (*n* = 2269)		
Age	21.30 ± 2.74	21.54 ± 2.89	21.17 ± 2.64	< 0.001	
Previous psychiatric diagnosis	219 (6.2%)	75 (6.0%)	144 (6.3%)	0.642	0.3%
Hours of Internet browsing per day	5.71 ± 4.47	5.57 ± 4.28	5.79 ± 4.58	0.153	
Alcohol use				0.125	
Not at all	2552 (72.3%)	878 (69.7%)	1674 (73.8%)		4.1%
One or 2 days	737 (20.9%)	284 (22.5%)	453 (20.0%)		2.5%
Several days	186 (5.3%)	76 (6.0%)	110 (4.8%)		1.2%
More than half the days	34 (1.0%)	14 (1.1%)	20 (0.9%)		0.2%
Nearly every day	20 (0.6%)	8 (0.6%)	12 (0.5%)		0.1%
Marihuana use				0.006	
Not at all	3334 (94.5%)	1166 (92.5%)	2168 (95.5%)		3.0%
One or 2 days	117 (3.3%)	55 (4.4%)	62 (2.7%)		1.7%
Several days	45 (1.3%)	22 (1.7%)	23 (1.0%)		0.7%
More than half the days	11 (0.3%)	6 (0.5%)	5 (0.2%)		0.3%
Nearly every day	22 (0.6%)	11 (0.9%)	11 (0.5%)		0.4%
Other substances use	728 (20.6%)	222 (17.6%)	506 (22.3%)	0.001	4.7%
GAD-7 score	6.17 ± 4.96	5.78 ± 4.93	6.39 ± 4.97	< 0.001	
PHQ-9 score	7.98 ± 5.63	7.62 ± 5.58	8.19 ± 5.65	0.004	
ICMH-IGD total sample score	13.30 ± 6.03	15.45 ± 6.81	12.11 ± 5.19	< 0.001	
Croatian sample score	437 (12.4%)	104 (8.3%)	333 (14.7%)	< 0.001	6.4%
Serbian sample score	316 (9.0%)	91 (7.2%)	225 (9.9%)	0.007	2.7%
Portuguese sample score	126 (3.6%)	31 (2.5%)	95 (4.2%)	0.008	1.7%
Turkish sample score	244 (6.9%)	94 (7.5%)	150 (6.6%)	0.341	0.9%
Vietnamese sample score	426 (12.1%)	141 (11.2%)	285 (12.6%)	0.231	1.4%
Polish sample score	156 (4.4%)	43 (3.4%)	113 (5.0%)	0.030	1.6%
Indonesian sample score	673 (19.1%)	188 (14.9%)	485 (21.4%)	< 0.001	6.5%
English sample score	1151 (32.6%)	568 (45.1%)	583 (25.7%)	< 0.001	19.4%

*p*-value calculated Student’s *t*-test for continuous variables and the *χ*^2^ test or Fisher’s exact test for categorical variables

*GAD-7* Generalized Anxiety Disorder scale-7, *PHQ-9* Patient Health Questionnaire-9, *ICMH-IGD* Internet Gaming Disorder Self-report for College/University Students by the International Child Mental Health Study Group

Table 2 shows the multiple linear regression analyses for males, which revealed that the total number of hours of Internet browsing per day (*β* = 0.14), anxiety symptoms (*β* = 0.09), and depression symptoms (*β* = 0.17) were significantly associated with more significant IGD symptoms, predicting 16.1% of the variance, when adjusted for culture and previous psychiatric diagnosis.

**Table 2:** Significant determinants of Internet gaming disorder self-report for college/university students by the International Child Mental Health Study Group, ICMH-IGD Scales, in 1260 male students

		ICMH-IGD score		PHQ-9 score		GAD-7 score		Alcohol use		Marihuana use	
		***β***		***β***		***β***		***β***		***β***	
**Model 1**	***R***^**2**^		0.007**		0.008**		0.005*		0.034***		0.000
	**Δ*R***^**2**^		0.007**		0.008**		0.005*		0.034***		0.000
Age		− 0.08**		− 0.09**		− 0.07*		0.19***		0.02	
**Model 2**	***R***^**2**^		0.096***		0.094***		0.092***		0.161***		0.039***
	**Δ*R***^**2**^		0.089***		0.086***		0.088***		0.127***		0.039***
Age		− 0.04		− 0.08**		− 0.08**		0.13***		− 0.01	
Previous psychiatric diagnosis		0.04		0.18***		0.22***		0.09***		0.06*	
Hours of Internet browsing per day		0.19***		0.16***		0.17***		0.11***		0.04	
**Model 3**	***R***^**2**^		0.161***		0.546***		0.544***		0.255***		0.153***
	**Δ*R***^**2**^		0.065***		0.452***		0.452***		0.094***		0.114***
Age		− 0.02		− 0.02		− 0.03		0.13***		− 0.03	
Previous diagnosis		− 0.02		0.02		0.09***		0.07*		0.01	
Hours of Internet browsing per day		0.14***		0.04		0.05*		0.09**		0.02	
Alcohol use		0.04		− 0.01		0.01				0.30***	
Marihuana use		0.05		0.01		0.03		0.26***			
Other substances		0.05		0.06**		0.04		0.13***		0.13***	
GAD-7 score		0.09**		0.69***				0.01		0.05	
PHQ-9 score		0.17***				0.70***		0.02		0.03	

*GAD-7* Generalized Anxiety Disorder scale-7, *PHQ-9* Patient Health Questionnaire-9, *ICMH-IGD* Internet Gaming Disorder Self-report for College/University Students by the International Child Mental Health Study Group

*p value <.05; **p value <.01; ***p value <.001 adjusted for culture

Table 3 shows the multiple linear regression analyses for females, which revealed that the total number of hours of Internet browsing per day (*β* = 0.13), marihuana use (*β* = 0.10), and depression symptoms (*β* = 0.21) were significantly associated with more significant IGD symptoms, predicting 17.6% of the variance, when adjusted for culture and previous psychiatric diagnosis.

**Table 3:** Significant determinants of Internet gaming disorder self-report for college/university students by the International Child Mental Health Study Group, ICMH-IGD Scales, in 2269 female students

		ICMH-IGD score		PHQ-9 score		GAD-7 score		Alcohol use		Marihuana use	
		***β***		***β***		***β***		***β***		***β***	
**Model 1**	***R***^**2**^		0.016**		0.013***		0.003*		0.018***		0.001
	**Δ*R***^**2**^		0.016**		0.013***		0.003*		0.018***		0.001
Age		− 0.13***		− 0.12***		− 0.06*		0.13***		0.03	
**Model 2**	***R***^**2**^		0.094***		0.112***		0.088***		0.176***		0.037
	**Δ*R***^**2**^		0.078***		0.098***		0.085***		0.158***		0.036***
Age		− 0.09***		− 0.13***		− 0.10***		0.01		− 0.03	
Previous psychiatric diagnosis		0.04		0.24***		0.19***		0.05*		0.05*	
Hours of Internet browsing per day		0.17***		0.15***		0.13***		0.04		0.05*	
**Model 3**	***R***^**2**^		0.176***		0.588***		0.576***		0.300***		0.185***
	**Δ*R***^**2**^		0.082***		0.476***		0.488***		0.124***		0.148***
Age		− 0.06**		− 0.05***		− 0.01		0.02		− 0.02	
Previous diagnosis		− 0.03		0.10***		0.01		0.01		0.02	
Hours of Internet browsing per day		0.13***		0.06***		0.02		0.02		0.03	
Alcohol use		0.06*		0.03		− 0.01				0.39***	
Marihuana use		0.10***		0.01		0.002		0.33***			
Other substances		0.05*		0.04**		0.04**		0.08***		0.12***	
GAD-7		0.05		0.71***				− 0.02		0.02	
PHQ-9		0.21***				0.73***		0.05		0.02	

*GAD-7* Generalized Anxiety Disorder scale-7, *PHQ-9* Patient Health Questionnaire-9, *ICMH-IGD* Internet Gaming Disorder Self-report for College/University Students by the International Child Mental Health Study Group

*p value <.05; **p value <.01; ***p value <.001 adjusted for culture

### Structural equation models

**Fig. 1:**
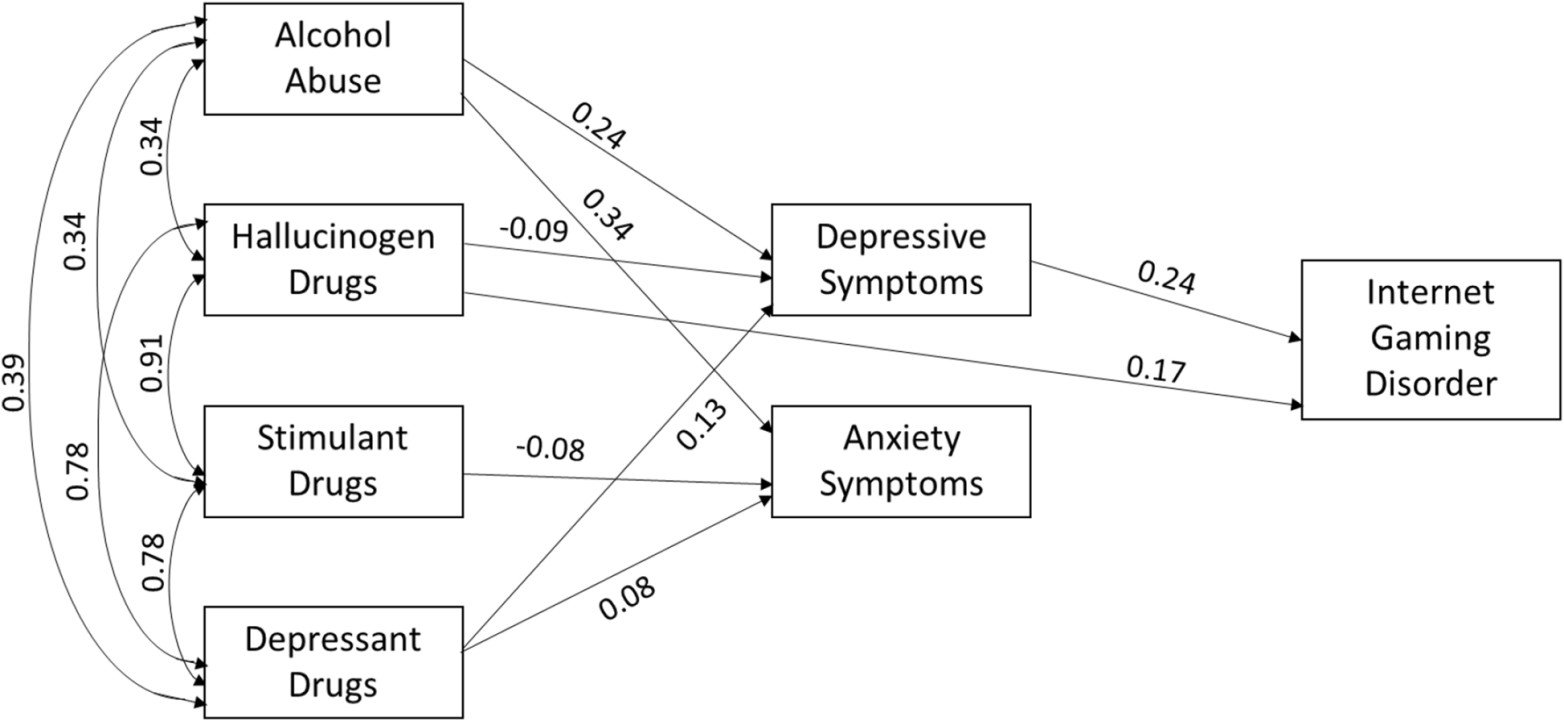
Relationships between alcohol abuse, stimulant, depressant, and hallucinogens drugs, with depressive, anxiety, and IGD symptoms in the whole sample

The model shown in Fig. 1 examined the relationships between alcohol abuse, stimulant drugs, depressant drugs, hallucinogens drugs, depressive, anxiety, and IGD symptoms in the whole sample. Alcohol abuse was positively associated depressive (*β* = 0.24, CI95% [0.21; 0.27], *p* < 0.001) and anxiety symptoms (*β* = 0.34, CI [0.31; 0.37], *p* < 0.001). Depressant drugs were positively associated with depressive (*β* = 0.13, CI95% [0.8; 0.19], *p* < 0.001) and anxiety symptoms (*β* = 0.08, CI95% [0.03; 0.13], *p* = 0.004). Stimulant drugs were negatively associated with anxiety symptoms (*β* = -0.08, CI95% [− 0.13; − 0.02], *p* = 0.002), hallucinogen drugs with depressive symptoms (*β* = − 0.09, CI95% [− 0.14; − 0.03], *p* = 0.002), and with IGD symptoms (*β* = 0.17, CI95% [0.14; 0.21], *p* < 0.001). Depressive symptoms were positively associated with IGD symptoms (*β* = 0.24, CI95% [0.20; 0.27], *p* < 0.001).

**Fig. 2:**
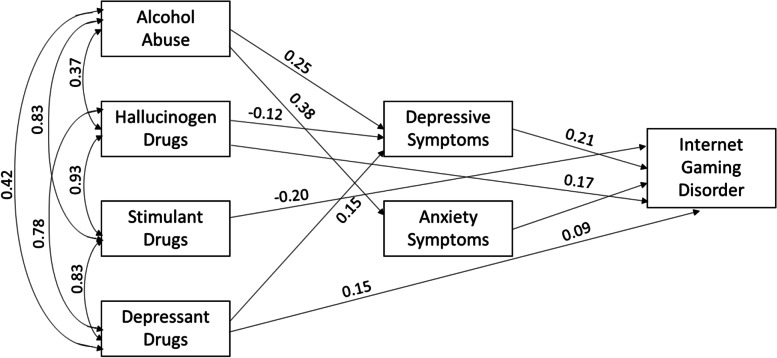
Relationships between alcohol abuse, stimulant, depressant, and hallucinogens drugs, with depressive, anxiety, and IGD symptoms in males

The model shown in Fig. 2 examined the relationships between alcohol abuse, stimulant drugs, depressant drugs, hallucinogens drugs, depressive, anxiety, and IGD symptoms in males. Alcohol abuse was positively associated depressive (*β* = 0.25, CI95% [0.19; 0.30], *p* < 0.001) and anxiety symptoms (*β* = 0.38, CI95% [0.34; 0.43], *p* < 0.001). Depressant drugs were positively associated with depressive symptoms (*β* = 0.15, CI95% [0.06; 0.25], *p* < 0.001), and IGD (*β* = 0.15, CI95% [0.05; 0.26], *p* = 0.004). Stimulant drugs were negatively associated with IGD symptoms (*β* = -0.20, CI95% [− 0.35; − 0.05], *p* = 0.002), hallucinogen drugs with depressive symptoms (*β* = − 0.12, CI95% [− 0.21; − 0.03], *p* = 0.012), but positively associated with IGD (*β* = 0.17, CI95% [0.02; 0.32], *p* = 0.022). Depressive symptoms were positively associated with IGD symptoms (*β* = 0.21, CI95% [0.13; 0.28], *p* < 0.001) as well as anxiety symptoms (*β* = 0.09, CI95% [0.02; 0.17], *p* = 0.01).

**Fig. 3:**
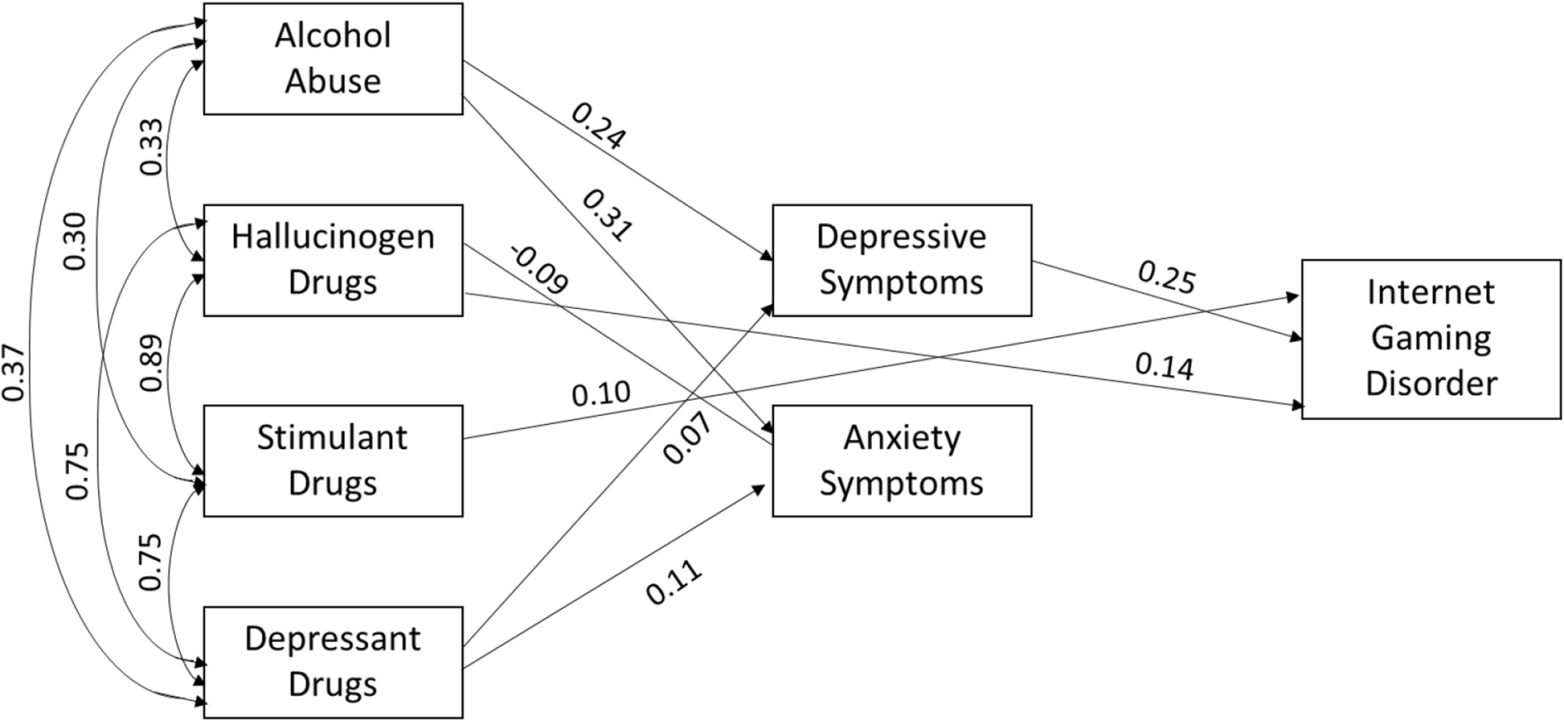
Relationships between alcohol abuse, stimulant, depressant, and hallucinogens drugs, with depressive, anxiety, and IGD symptoms in females

The model shown in Fig. 3 examined the relationships between alcohol abuse, stimulant drugs, depressant drugs, hallucinogens drugs, depressive, anxiety, and IGD symptoms in females. Alcohol abuse was positively associated depressive (*β* = 0.24, CI95% [0.19; 0.28], *p* < 0.001) and anxiety symptoms (*β* = 0.31, CI95% [0.27; 0.35], *p* < 0.001). Depressant drugs were positively associated with depressive (*β* = 0.07, CI95% [0.03; .11], *p* < 0.001) and anxiety symptoms (*β* = 0.11, CI95% [0.05; 0.17], *p* < 0.001). Stimulant drugs were positively associated with IGD symptoms (*β* = -0.10, CI95% [− 0.35; -0.05], *p* = 0.002). Hallucinogen drugs were negatively associated with anxiety (*β* = − 0.08, CI95% [− 0.15; − 0.03], *p* = 0.004), but positively with IGD symptoms (*β* = 0.14, CI95% [0.06; 0.23], *p* < 0.001). Depressive symptoms were positively associated with IGD symptoms (*β* = 0.25, CI95% [0.21; 0.28], *p* < 0.001).

## Discussion

In this international, multicultural sample of young adults, we found that anxiety and depressive symptoms and substance use were associated with IGD symptoms independently of Internet use time, presence of psychiatric diagnosis, culture, or sociodemographic characteristics. Importantly, we found gender differences in the observed associations. Besides extended Internet browsing per day for both, more significant IGD symptoms were associated additionally with higher levels of anxiety and depressive symptoms for males, while with marihuana use and higher levels of depressive symptoms for females.

Our study confirmed a higher presence of IGD symptomatology in males compared to females as previously reported [32]. We also showed a differential pattern of associations of IGD symptomatology with anxiety and depressive symptoms and substance use in males and females. In this way, we confirmed a previous study [38] that in a smaller sample found both high anxiety and depressive symptoms to be associated with greater IGD symptoms, where greater symptomatology of IGD in males compared to females, while in females, only depressive symptoms were associated with IGD. Despite the general contributing factors to IGD symptomatology, we observed that IGD symptoms were differently affected by various substance use in males and females. The general association of IGD and substance use is in line with a recent review [28], which concluded that many of the problematic behaviors co-occurring with disordered gaming can be performed concurrently with problematic gaming, potentially as part of a coping strategy [45]. Unique associations between various substance use, psychological symptoms, and IGD found in our study for males and females might indicate possible gender-specific predisposition, coping strategies, or vulnerabilities, what may be used in therapeutic approaches to tackle IGD symptom severity as gender specific. These gender differences suggest that IGD may be associated with a “stimulation” experience of using substances in females (as revealed by a positive association between stimulant drugs and absence of association between depressant drugs and IGD symptoms) and a “depressive” in males (as revealed by the negative association between stimulant drugs and IGD symptoms and a positive association between depressant drugs and IGD symptoms). This is consistent with evidence showing that actions occurring for natural rewards and that there are common and specific underpinnings for the associations between drug use and gaming disorder. Future studies that clarify neurobiology [46] and coping strategies [45] explain gender differences in IGD. The available literature suggests the possible gender-specific association between cannabis use and depressive symptoms, with females showing stronger associations between cannabis use and depression than males [47]. However, surveys consistently show that anxiety disorders are more common among females [48] and females may be more prone than males to self-medicate for mood problems with substances such as alcohol [49]; moreover, females are more likely to drink to regulate negative affect and stress reactivity [50, 51]. Our findings suggest that specific drug use may strengthen the impact of symptoms of anxiety and depression on IGD. These patterns of associations need to be studied further; however, they are in line with the current knowledge on differences in gender-specific mechanisms of alcohol and cannabis effects. Encountering substance use severity and its relationship to the prevalence of anxiety/depression symptoms is an important future direction for developing gender-appropriate interventions to address IGD.

The results of our study should be interpreted with caution due to some relevant limitations. First, we evaluated the relationship between symptoms of anxiety and depression only on IGD. An association of IGD/problematic Internet use and impulsivity is frequently reported [52–54]. For example, Ryu et al. [55] showed that high impulsivity affected depressive symptoms and increased the risk of IGD. Thus, future studies should evaluate impulsivity alongside symptoms of anxiety and depression. Whether the currently observed effect is the same in other types of stress-related conditions, personality traits [56], emotional intelligence [15], or other traits/motives such as escapism [57] is unknown. Another major limitation is that we used a cross-sectional study design, and to demonstrate causal effects, prospective studies are needed and clinical trials on the interventions addressing both substance use and anxiety and depression management. In addition, only students who agreed to participate were included, and that the response rates varied substantially between countries. Furthermore, the sampling was convenient, and those who may have different or more pronounced patterns of IGD might not have participated. In addition, we equated a country-level with culture-level, which may not be sufficient to represent culture, and we could not obtain enough data for other sub-cultural and sub-racial groups, and we were not collecting data on Internet access availability, equipment used, and legal conditions of some substances’ availability, which could limit the generalizability of the findings. Finally, only self-reports were used to assess psychiatric symptoms, and respective diagnoses were not confirmed since it was not able to provide clinical assessments to the participants.

## Conclusions

Our study found that more overt symptoms of IGD were associated with higher levels of anxiety and depressive symptoms and substance use. Still, these associations may differ among males and females, what should be considered in therapeutic approaches targeting IGD. Factors mediating the relationship between mental distress and IGD symptoms might not be the same as contributing factors. Therefore, a better understanding of mechanisms related to psychiatric symptoms contributing to IGD symptoms is warranted.

## Supplementary Information

**Additional file 1.**

## Data Availability

The datasets used and/or analyzed during the current study are available from the corresponding author on reasonable request.

## Abbreviations

ICD: International Classification of Diseases
ICMH-SG: International Child Mental Health – Study Group
ICMH-IGD: ICMH-Internet Gaming Disorder Self-report for College/University Students
IGD: Internet gaming disorder
DSM: Diagnostic and Statistical Manual
GAD-7: Generalized Anxiety Disorder scale-7
PHQ-9: Patient Health Questionnaire-9
SEM: Structural equation modeling
UAE: United Arab Emirates
