# Supplementary material for: Association of Internet gaming disorder symptoms with anxiety and depressive symptoms and substance use: an international cross-sectional study
Source: Middle East Curr Psychiatry. 2022 Feb 14;29(1):14. doi: 10.1186/s43045-022-00180-6 (PMC8853349; doi:10.1186/s43045-022-00180-6)
Supplement: Supplementary file 1 — Additional file 1. [file 43045_2022_180_MOESM1_ESM.docx]

| **Supplementary Material**  *Substance use over the past month* | | | | | | | | | | | | | | | | | | | | | | | | |
| --- | --- | --- | --- | --- | --- | --- | --- | --- | --- | --- | --- | --- | --- | --- | --- | --- | --- | --- | --- | --- | --- | --- | --- | --- |
|  | Stimulants | | Sedatives or tranquilizers | | Alcohol | | Marihuana | | | Cocaine or crack | | Club Drugs | | | Hallucinogens | | Heroin | | Inhalants or solvents | | Methamphetamines | | Painkillers | |
|  | N | % | N | % | N | % | | N | % | N | % | | N | % | N | % | N | % | N | % | N | % | N | % |
| Not at all | 3450 | 97.8 | 3327 | 94.3 | 2552 | 72.3 | | 3334 | 94.5 | 3462 | 98.1 | | 3466 | 98.2 | 3470 | 98.3 | 3474 | 98.4 | 3462 | 98.1 | 3465 | 98.2 | 2934 | 83.1 |
| One or two days | 52 | 1.5 | 133 | 3.8 | 737 | 20.9 | | 117 | 3.3 | 32 | .9 | | 29 | .8 | 24 | .7 | 24 | .7 | 36 | 1.0 | 29 | .8 | 476 | 13.5 |
| Several days | 13 | .4 | 49 | 1.4 | 186 | 5.3 | | 45 | 1.3 | 22 | .6 | | 18 | .5 | 20 | .6 | 15 | .4 | 18 | .5 | 21 | .6 | 78 | 2.2 |
| More than half of the days | 1 | .0 | 9 | .3 | 34 | 1.0 | | 11 | .3 | 4 | .1 | | 7 | .2 | 5 | .1 | 10 | .3 | 5 | .1 | 9 | .3 | 26 | .7 |
| Nearly every day | 13 | .4 | 11 | .3 | 20 | .6 | | 22 | .6 | 9 | .3 | | 9 | .3 | 10 | .3 | 6 | .2 | 8 | .2 | 5 | .1 | 15 | .4 |

| *Use of Stimulant drugs* | | | | |
| --- | --- | --- | --- | --- |
|  | | Stimulants | Cocaine or crack | Methamphetamines |
| Stimulants | Stimulants | 34 | --- | --- |
|  | Cocaine or crack | 5 | --- | --- |
|  | Methamphetamines | 5 | --- | --- |
| Cocaine or crack | Stimulants |  | 5 | 35 |
|  | Cocaine or crack |  | 16 | --- |
|  | Methamphetamines | 35 | 11 | --- |
| Methamphetamines | Stimulants |  | 35 | 5 |
|  | Cocaine or crack | --- | --- | 11 |
|  | Methamphetamines | --- | --- | 13 |
| Total | | 79 | 67 | 64 |

| *Use of Depressant Drugs* | | | | | | | |
| --- | --- | --- | --- | --- | --- | --- | --- |
| Painkillers | Inhalants or solvents | Heroin | | | Marihuana | | Total |
|  |  |  |  |  | Do not use | Use at least one or two days |  |
| Do not use | Do not use | Do not use | Sedatives or tranquillizers | Do not use | 2742 | 99 | 2841 |
|  |  |  |  | Use at least one or two days | 70 | 8 | 78 |
|  |  | Use at least one or two days | Sedatives or tranquillizers | Do not use | 1 | 1 | 2 |
|  |  |  |  | Use at least one or two days | 0 | 0 | 0 |
|  | Use at least one or two days | Do not use | Sedatives or tranquillizers | Do not use | 4 | 0 | 4 |
|  |  |  |  | Use at least one or two days | 2 | 0 | 2 |
|  |  | Use at least one or two days | Sedatives or tranquillizers | Do not use | 0 | 1 | 1 |
|  |  |  |  | Use at least one or two days | 2 | 4 | 6 |
| Use at least one or two days | Do not use | Do not use | Sedatives or tranquillizers | Do not use | 431 | 36 | 467 |
|  |  |  |  | Use at least one or two days | 63 | 6 | 69 |
|  |  | Use at least one or two days | Sedatives or tranquillizers | Do not use | 3 | 0 | 3 |
|  |  |  |  | Use at least one or two days | 1 | 1 | 2 |
|  | Use at least one or two days | Do not use | Sedatives or tranquillizers | Do not use | 7 | 0 | 7 |
|  |  |  |  | Use at least one or two days | 3 | 3 | 6 |
|  |  | Use at least one or two days | Sedatives or tranquillizers | Do not use | 0 | 2 | 2 |
|  |  |  |  | Use at least one or two days | 5 | 34 | 39 |
| Total |  |  |  |  | 3334 | 195 | 3529 |

| *Use of Hallucinogen drugs* | | |
| --- | --- | --- |
|  | Club Drugs | Hallucinogens |
| Club Drugs | 16 | 47 |
| Hallucinogens | 47 | 12 |
| Total | 63 | 59 |
